# The prognostic value of the neutrophil percentage-to-albumin ratio for all-cause and cardiovascular mortality in chronic kidney disease: A retrospective cohort study using NHANES 1999 to 2018

**DOI:** 10.1097/MD.0000000000049786

**Published:** 2026-07-17

**Authors:** Rong Ni, Haixia Zhang, Linsen Jiang, Yusheng Xu, Ci Sun, Zhijian Zhang, Weiwei Li

**Affiliations:** a Department of Nephrology, The Second Affiliated Hospital of Soochow University, Suzhou, China; b Department of Nephrology, The Affiliated Wuxi People’s Hospital of Nanjing Medical University, Wuxi People’s Hospital, Wuxi Medical Center, Nanjing Medical University, Wuxi, Jiangsu, China.

**Keywords:** all-cause mortality, cardiovascular mortality, chronic kidney disease, neutrophils, serum albumin

## Abstract

Chronic kidney disease (CKD) is a global public health challenge associated with elevated all-cause and cardiovascular mortality, driven by chronic inflammation and malnutrition. The neutrophil percentage-to-albumin ratio (NPAR), integrating inflammatory, and nutritional markers, has shown prognostic value in other conditions but remains underexplored in CKD. This study aimed to evaluate the association between NPAR and mortality outcomes in CKD patients using a nationally representative cohort. Data from National Health and Nutrition Examination Survey 1999 to 2018 were analyzed, including 1990 adults with CKD. NPAR was calculated as neutrophil percentage divided by serum albumin and categorized into quartiles. Primary outcomes were all-cause and cardiovascular mortality, assessed via linkage with the National Death Index. Cox regression models adjusted for demographics, comorbidities, and laboratory parameters. Restricted cubic splines and mediation analyses explored dose–response relationships and underlying mechanisms. Higher NPAR quartiles were significantly associated with increased all-cause mortality (Q4 vs Q1: adjusted hazard ratio = 2.04, 95% confidence interval = 1.41–3.00, *P* < .001) and cardiovascular mortality (Q4 vs Q1: hazard ratio = 2.33, 95% confidence interval = 1.20–4.50, *P* = .012). Kaplan–Meier analysis revealed poorer survival in Q4 (*P* < .001). Subgroup analyses showed stronger NPAR-mortality associations in obese, hypertensive, and cardiovascular disease patients. Mediation analysis indicated no significant role of estimated glomerular filtration rat in these relationships. NPAR is an independent predictor of all-cause and cardiovascular mortality in CKD, particularly in high-risk subgroups. This biomarker may enhance risk stratification by reflecting combined inflammatory and nutritional dysfunction, supporting targeted interventions to improve outcomes. Further prospective studies are needed to validate its clinical utility.

## 1. Introduction

Chronic kidney disease (CKD) affects approximately 9.1% of the global population (~700 million in 2017) and is associated with rising mortality and disability-adjusted life years, representing a major public health burden.^[1]^ Epidemiological studies indicate that CKD patients exhibit substantially elevated all-cause and cardiovascular mortality, with nearly 50% of deaths attributable to cardiovascular disease (CVD).^[2–4]^ This poor prognosis stems from the complex interplay of chronic inflammation, malnutrition, and accelerated atherosclerosis, collectively termed the “malnutrition-inflammation-atherosclerosis (MIA) syndrome.”^[5]^ Among these pathophysiological mechanisms, systemic inflammation and protein-energy wasting have been identified as key drivers of disease progression and adverse outcomes.^[6–8]^

The neutrophil percentage-to-albumin ratio (NPAR) is a novel composite biomarker that integrates neutrophilic inflammation (reflected by neutrophil percentage) and nutritional status (indicated by serum albumin), offering a comprehensive tool for risk stratification in CKD.^[9]^ Neutrophils, as primary effectors of innate immunity, exacerbate systemic inflammation through oxidative stress and cytokine release, whereas hypoalbuminemia reflects both inflammation-mediated suppression of hepatic synthesis and malnutrition. Emerging evidence suggests that NPAR predicts mortality in conditions such as sepsis,^[10,11]^ acute coronary syndromes,^[12,13]^ and cancer.^[14–16]^ For instance, a 2023 study by Wang et al^[17]^ demonstrated that NPAR serves as a robust predictor for both short-term and long-term all-cause mortality in patients with heart failure. However, despite its strong mechanistic relevance to CKD pathophysiology, the prognostic value of NPAR in CKD remains underexplored, particularly regarding its association with hard endpoints such as cardiovascular mortality.

To investigate this, we analyzed data from the US National Health and Nutrition Examination Survey (NHANES 1999–2018) to evaluate the association between NPAR and mortality outcomes in a nationally representative CKD cohort. By leveraging this large-scale database, we aimed to assess the predictive value of NPAR for mortality in CKD. Our findings may provide clinicians with an easily measurable biomarker to identify high-risk patients, thereby guiding early interventions targeting inflammation and nutritional support.

## 2. Methods

### 2.1. Study population

**Figure 1.**
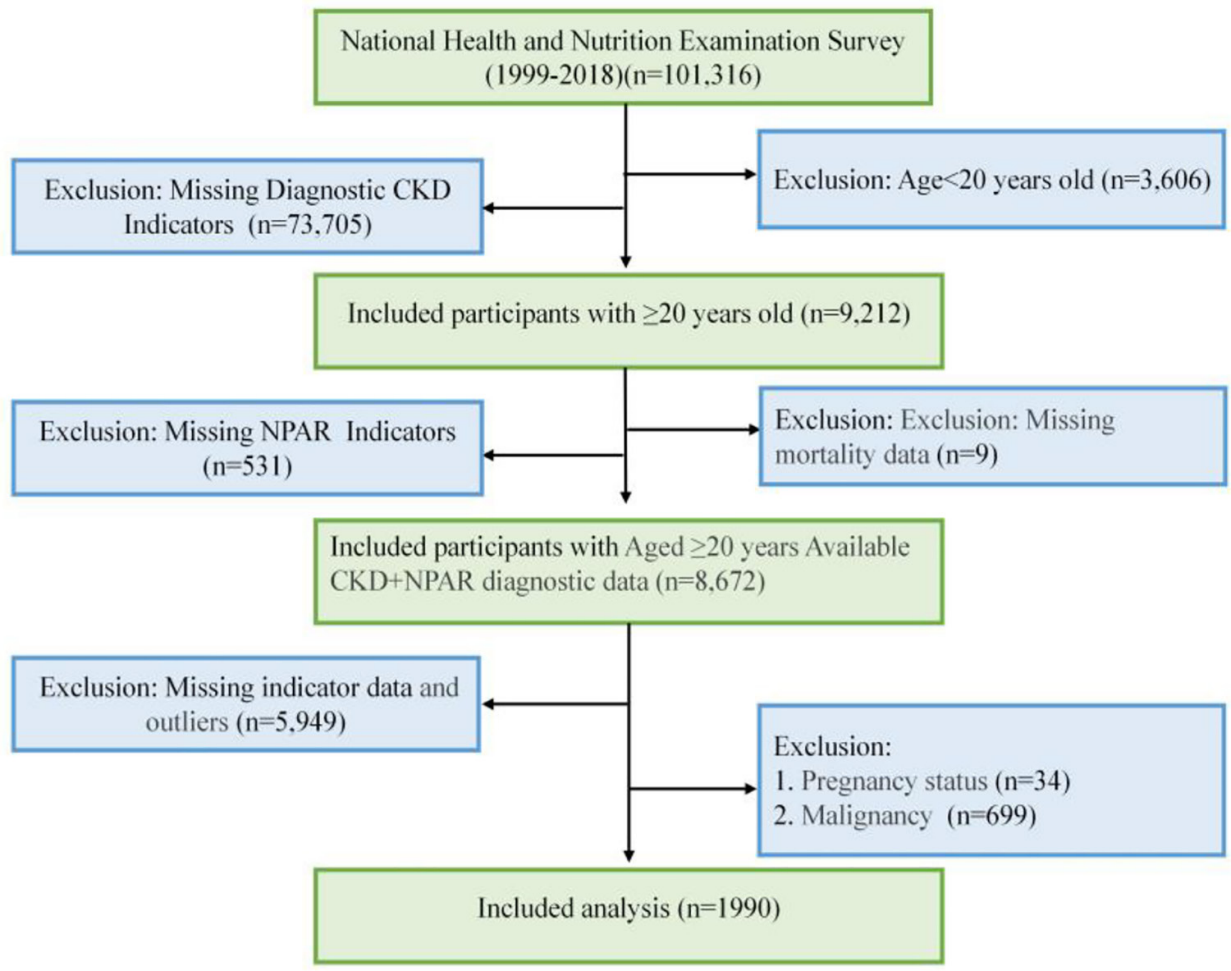
Screening flow of respondents. CKD = chronic kidney disease, NPAR = neutrophil percentage-to-albumin ratio.

This analysis utilized data from the NHANES (1999–2018), a nationally representative cross-sectional study conducted by the Centers for Disease Control and Prevention. The survey used a complex, multistage, stratified probability sampling design to obtain health and nutritional parameters from the noninstitutionalized civilian population of the United States. Inclusion criteria comprised: participants aged 20 years or older; completion of all required survey components, including standardized questionnaires, physical examinations, and laboratory assessments; availability of complete data for NPAR calculation; and documented CKD diagnostic parameters and complete covariate data. To enhance data quality and minimize potential confounding, we implemented the following exclusion criteria: individuals younger than 20 years; participants with any history of malignant neoplasms; women who were pregnant at the time of examination; and cases with incomplete mortality follow-up data or missing event documentation. The final analytical cohort (n = 1990) demonstrated representative characteristics of the US adult population with CKD. Figure 1 presents the detailed participant selection flowchart. All analyses incorporated appropriate sampling weights and accounted for the complex survey design to ensure national representativeness and validity of statistical inferences.

### 2.2. Definition of CKD

CKD diagnosis was established based on internationally recognized criteria using NHANES laboratory and clinical data. The diagnosis required fulfillment of either of the following criteria: renal function assessment: estimated glomerular filtration rate (eGFR) was calculated using the chronic kidney disease epidemiology collaboration 2009 creatinine equation with race adjustment^[18]^: for serum creatinine (Scr) ≤0.7 mg/dL in females or ≤0.9 mg/dL in males: eGFR = 144 × (Scr/0.7)^−0.329 × 0.993^age × 1.159 (if African American), for Scr >0.7 mg/dL in females or >0.9 mg/dL in males: eGFR = 144 × (Scr/0.7)^−1.209 × 0.993^age × 1.159 (if African American). Persistent eGFR <60 mL/min/1.73 m^2^ for ≥3 months confirmed CKD diagnosis; albuminuria evaluation: urinary albumin-to-creatinine ratio (UACR) was measured from first-morning void specimens. Albuminuria was defined as UACR ≥30 mg/g. CKD staging was performed according to Kidney Disease: Improving Global Outcomes 2012 guidelines^[19]^: stage 1: eGFR ≥90 mL/min/1.73 m^2^ with UACR ≥30 mg/g, stage 2: eGFR 60–89 mL/min/1.73 m^2^ with UACR ≥30 mg/g, stage 3: eGFR 30–59 mL/min/1.73 m^2^, stage 4: eGFR 15–29 mL/min/1.73 m^2^, and stage 5: eGFR <15 mL/min/1.73 m^2^.Given the cross-sectional nature of NHANES, CKD status was defined based on a single assessment of eGFR and/or UACR, consistent with prior NHANES-based studies. Persistent CKD requiring confirmation over ≥3 months could not be ascertained in this study.

### 2.3. Measurement of NPAR

NPAR was calculated using the same blood sample and the following formula^[20]^: neutrophil percentage (in total white blood cell count) (%) × 100/albumin (g/dL). Currently, there is no established classification standard for NPAR. In this study, NPAR values were categorized into quartiles: Q1: NPAR < 12.51, Q2: 12.51 ≤ NPAR < 14.27, Q3: 14.27 ≤ NPAR < 15.70, and Q4: NPAR ≥ 15.70.

### 2.4. Primary outcomes

The primary endpoints were cardiovascular mortality and all-cause mortality, ascertained through linkage with the National Death Index via the NHANES Public-Use Linked Mortality File (follow-up through December 31, 2019). All-cause mortality encompassed deaths from any cause during follow-up. Cardiovascular mortality was defined as fatal events attributable to CVD, including coronary artery disease (I20–I25), heart failure (I50), cerebrovascular events (I60–I69), and other circulatory disorders (ICD-10 codes I00–I99). Follow-up duration (in months) was calculated from baseline assessment to either the recorded date of death or the study termination date (December 31, 2019) for surviving participants.

### 2.5. Variable assessment

This comprehensive analysis incorporated multiple variable categories from the NHANES database, including demographic factors, clinical measurements, laboratory data, and behavioral covariates. All data were collected through standardized protocols by trained personnel: demographic characteristics: age (continuous), sex (male/female), and race/ethnicity (Mexican American, Hispanic, non-Hispanic White, non-Hispanic Black, other), education attainment (categorized as: <high school, high school/equivalent, ≥college), and socioeconomic status (poverty income ratio: family income to poverty threshold ratio); clinical parameters: anthropometrics: body mass index (BMI), systolic blood pressure, diastolic blood pressure (DBP), comorbidities: self-reported physician-diagnosed hypertension, diabetes, and CVD; behavioral factors: smoking status: never (<100 lifetime cigarettes), former (≥100 cigarettes, currently abstinent), and current (active use at survey). Alcohol consumption (standard drinks/day; 1 drink = 14 g ethanol): mild (women ≤1, men ≤2 drinks/day), moderate (women 1–3, men 2–4 drinks/day), and heavy (women ≥4, men ≥5 drinks/day); and laboratory measurements: all biochemical analyses were performed at mobile examination centers by certified technicians following strict quality control protocols.

### 2.6. Statistical analysis

All statistical analyses were conducted using R software (version 4.3.3; R Foundation for Statistical Computing) and GraphPad Prism 9 (GraphPad Software) for forest plot generation. On the basis of NPAR values, the study cohort was stratified into quartiles (Q1–Q4). Descriptive statistics were computed for demographic characteristics (age, sex, race, and marital status), lifestyle factors (smoking history, alcohol consumption), and clinical laboratory parameters. Continuous variables with normal distribution were expressed as mean ± standard deviation and compared using one-way analysis of variance; non-normally distributed variables were reported as median (interquartile range) and analyzed using the Kruskal–Wallis test. Categorical variables were presented as frequencies (percentages) and compared via chi-square or Fisher exact test, as appropriate. Survival outcomes were assessed using Kaplan–Meier analysis, with differences between NPAR quartiles evaluated by the log-rank test. Survival time was calculated from enrollment until the occurrence of cardiovascular death or all-cause death, with censoring applied for loss to follow-up or end of study. Univariate and multivariate Cox proportional hazards regression models were used to determine the independent association between NPAR and mortality outcomes. Hazard ratios (HRs) with 95% confidence intervals (CIs) were computed after adjusting for covariates, including demographics, comorbidities (e.g., diabetes, hypertension), and laboratory markers. Variable selection was performed in 2 stages: initial screening: variables showing significant between-group differences (*P* < .05 in baseline comparisons) were identified; and univariable Cox regression: only variables meeting the significance threshold (*P* < .05) in this preliminary analysis were retained for further multivariable modeling. The proportional hazards assumption was verified using Schoenfeld residuals. Restricted cubic splines were used to explore nonlinear relationships between NPAR and mortality in adjusted models. To enhance robustness, sensitivity analyses were performed: stratified analyses by age (<65 vs ≥65 years), sex, BMI, marital status, and comorbid conditions; and mediation analysis to quantify the proportion of NPAR’s effect mediated by eGFR, with bootstrapping (1000 iterations). All tests were two-sided, with *P* < .05 considered statistically significant.

### 2.7. Ethical approval and consent to participate

This study analyzed data from the NHANES, a publicly available de-identified dataset. The original NHANES protocol adhered to ethical principles outlined in the Declaration of Helsinki, with written informed consent obtained from all participants. All procedures were performed in accordance with institutional guidelines and federal regulations governing human subjects research.

## 3. Results

### 3.1. Baseline characteristics

**Figure 2.**
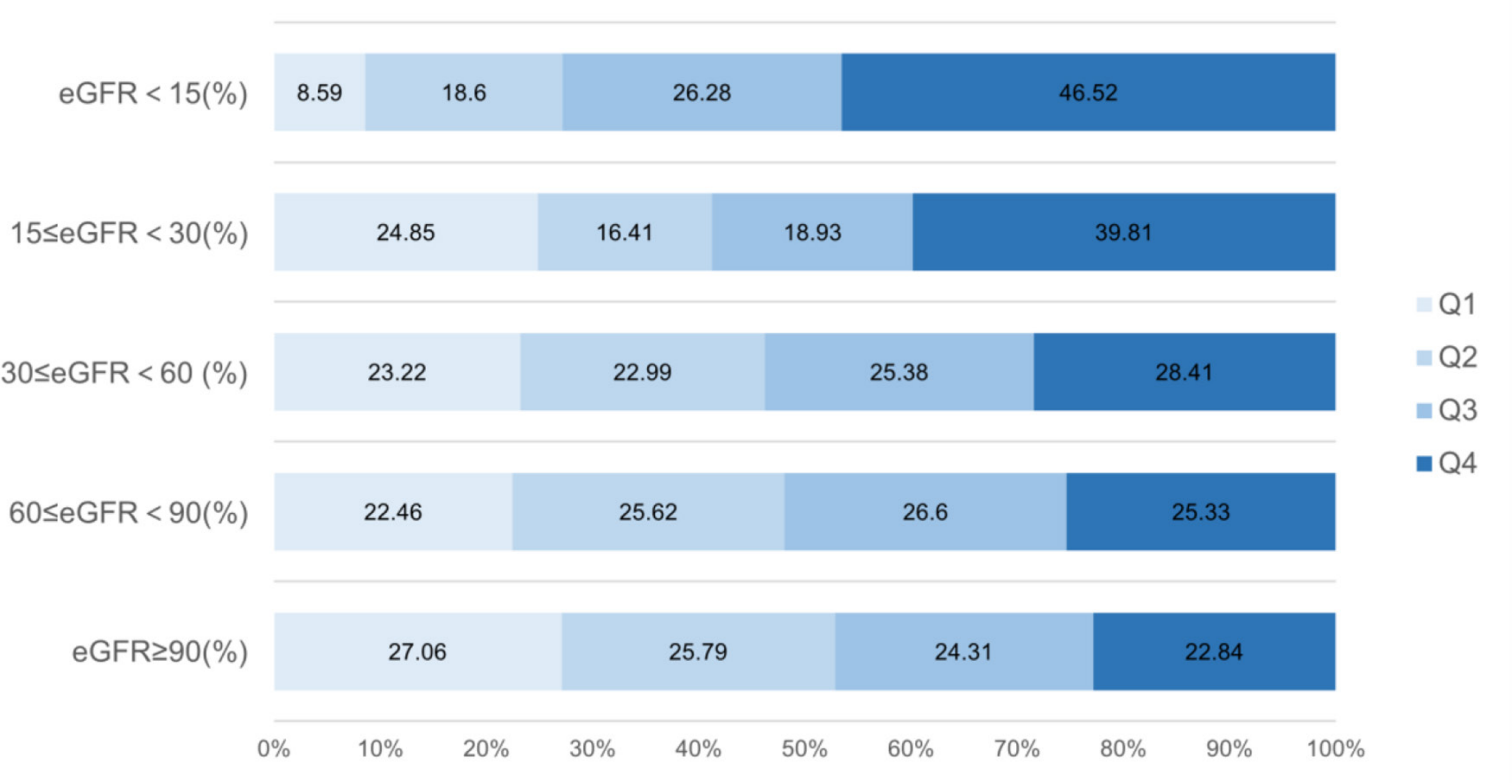
Distribution of eGFR categories by NPAR quartiles. Q1: NPAR < 12.51, Q2: 12.51 ≤ NPAR < 14.27, Q3: 14.27 ≤ NPAR < 15.70, Q4: NPAR ≥ 15.70. eGFR = estimated glomerular filtration rate, NPAR = neutrophil percentage-to-albumin ratio.

This study enrolled 1990 CKD patients, including 1076 males (48.56%) and 914 females (51.44%). The median age was 53 years (interquartile range: 39–67). Comorbid conditions included hypertension (n = 1292, 52.64%), diabetes mellitus (n = 664, 26.59%), and CVD (n = 382, 15.41%). During follow-up, 485 deaths (19.98%) were recorded, including 163 cardiovascular deaths (6.04%). Participants were stratified by NPAR quartiles: Q1 (NPAR < 12.51): 511 patients (25.68%); Q2 (12.51 ≤ NPAR < 14.27): 487 patients (24.47%); Q3 (14.27 ≤ NPAR < 15.70): 463 patients (23.27%); Q4 (NPAR ≥ 15.70): 529 patients (26.58%). Significant intergroup differences (*P* < .05) were observed in: age, race, smoking, physical activity, DBP, BMI, serum albumin, hemoglobin, total cholesterol, hypertension, diabetes, and CVD. No significant differences (*P* > .05) were found in: sex, marital status, education level, alcohol consumption, systolic blood pressure, poverty income ratio, serum uric acid, creatinine, UACR, eGFR (Table 1). A progressive increase in Q4 prevalence was observed with declining eGFR, rising from 22.84% to 46.52% (*P* < .001; Fig. 2).

**Table 1:** Baseline characteristics of patients with CKD according to quartiles of NPAR in NHANES (1999–2018).

Variable	Total (n = 1990)	Q1 (n = 511)	Q2 (n = 487)	Q3 (n = 463)	Q4 (n = 529)	*F*/χ^2^	*P* value
Age (yr)	53 (39–67)	50 (35–64)	53 (40–66)	56 (42–68)	55 (40–72)	13.488	.005
Sex, n (%)						5.856	.301
Male	1076 (48.56)	288 (49.20)	264 (52.44)	249 (47.67)	275 (44.93)		
Female	914 (51.44)	223 (50.81)	223 (47.56)	214 (52.33)	254 (55.07)		
Race, n (%)						49.896	<.001
Mexican American	286 (7.28)	61 (6.32)	79 (8.29)	73 (7.45)	73 (7.06)		
Other Hispanic	162 (5.15)	41 (4.94)	39 (4.31)	38 (4.38)	44 (6.96)		
Non-Hispanic White	918 (69.05)	181 (61.86)	210 (67.21)	252 (76.65)	275 (70.47)		
Non-Hispanic Black	504 (13.37)	183 (19.04)	131 (15.04)	82 (8.00)	108 (11.41)		
Other race	120 (5.15)	45 (7.84)	28 (5.15)	18 (3.53)	29 (4.09)		
Marriage, n (%)						4.060	.550
Yes	1594 (79.22)	400 (76.50)	393 (80.02)	373 (81.51)	428 (78.82)		
No	396 (20.79)	111 (23.50)	94 (19.98)	90 (18.49)	101 (21.18)		
PIR	3.05 (1.51–5.00)	3.37 (1.54–5.00)	3.23 (1.71–5.00)	3.05 (1.57–5.00)	2.58 (1.37–5.00)	5.933	.120
Smoking, n (%)						29.108	.019
Never	874 (45.57)	232 (48.89)	238 (51.38)	187 (42.76)	217 (39.28)		
Former	630 (30.89)	145 (26.75)	144 (29.46)	147 (29.53)	194 (37.81)		
Now	486 (23.54)	134 (24.37)	105 (19.16)	129 (27.71)	118 (22.91)		
Education, n (%)						9.269	.447
Less than high school	474 (16.25)	114 (16.61)	107 (16.71)	119 (14.95)	134 (16.73)		
High school or equivalent	487 (23.11)	125 (22.25)	144 (27.40)	116 (22.55)	102 (20.24)		
College or above	1029 (60.64)	272 (61.14)	236 (55.89)	228 (62.50)	293 (63.03)		
Drinking, n (%)						21.209	.078
Mild	1078 (52.10)	258 (47.54)	246 (48.23)	259 (52.87)	315 (59.73)		
Moderate	567 (31.41)	156 (35.73)	158 (34.39)	123 (30.88)	130 (24.63)		
Heavy	345 (16.50)	97 (16.73)	83 (17.38)	81 (16.25)	84 (15.64)		
Physical activity, n (%)						14.808	.044
Low physical activity	862 (37.90)	202 (35.14)	195 (33.33)	208 (38.85)	257 (44.28)		
High physical activity	1128 (62.10)	309 (64.86)	292 (66.67)	255 (61.15)	272 (55.72)		
SBP (mm Hg)	130.92 ± 0.64	130.10 ± 1.20	132.32 ± 1.46	130.28 ± 1.24	131.00 ± 1.28	0.522	.668
DBP (mm Hg)	72.93 ± 0.40	74.29 ± 0.73	74.35 ± 0.79	72.59 ± 0.79	70.50 ± 0.83	4.928	.003
BMI (kg/m^2^)	29.62 ± 0.25	27.73 ± 0.40	30.22 ± 0.54	29.19 ± 0.39	31.35 ± 0.50	11.155	<.001
Hypertension, n (%)						16.128	.042
No	698 (43.76)	207 (51.14)	154 (40.09)	166 (43.49)	171 (40.33)		
Yes	1292 (56.24)	304 (48.86)	333 (59.91)	297 (56.51)	358 (59.68)		
Diabetes, n (%)						31.564	<.001
No	1326 (73.41)	370 (81.20)	339 (76.06)	303 (69.17)	314 (67.23)		
Yes	664 (26.59)	141 (18.80)	148 (23.94)	160 (30.83)	215 (32.77)		
CVD, n (%)						27.536	<.001
No	1608 (84.59)	439 (90.51)	404 (85.99)	369 (83.00)	396 (78.88)		
Yes	382 (15.41)	72 (9.49)	83 (14.01)	94 (17.00)	133 (21.12)		
Albumin (g/L)	4.26 ± 0.01	4.42 ± 0.02	4.36 ± 0.02	4.27 ± 0.02	3.99 ± 0.02	111.251	<.001
Uric acid (mmol/L)	350.85 ± 3.41	346.35 ± 5.75	360.79 ± 7.28	345.73 ± 5.48	350.53 ± 6.13	1.135	.337
Creatinine (μmol/L)	80.44 (65.42–104.31)	81.33 (68.95–99.01)	79.60 (68.07–98.12)	81.33 (64.53–106.08)	81.33 (63.65–109.62)	0.598	.897
Hemoglobin (g/dL)	14.25 ± 0.05	14.33 ± 0.09	14.44 ± 0.08	14.33 ± 0.09	13.88 ± 0.09	9.661	<.001
Total cholesterol (mmol/L)	5.17 ± 0.040	5.37 ± 0.08	5.21 ± 0.07	5.17 ± 0.08	4.93 ± 0.05	9.768	<.001
UACR (mg/g)	44.93 (30.91–99.05)	41.71 (30.71–91.27)	44.43 (31.41–93.85)	47.09 (27.04–111.57)	47.83 (30.85–102.89)	4.274	.239
eGFR (mL/min/1.73 m^2^)	88.00 ± 1.013	91.18 ± 1.84	89.64 ± 1.81	86.88 ± 1.98	84.32 ± 1.89	2.589	.056

Normal distribution: mean ± SE, *P* values via weighted ANOVA, non-normal distribution: M (P25, P75), *P* values via weighted Kruskal–Wallis test.

Categorical: n, %, *P* values from weighted chi-square.

NPAR: neutrophil percentage-to-albumin ratio; Q1: NPAR < 12.51, Q2: 12.51 ≤ NPAR < 14.27, Q3: 14.27 ≤ NPAR < 15.70, Q4: NPAR ≥ 15.70.

ANOVA = analysis of variance, BMI = body mass index, CKD = chronic kidney disease, CVD = cardiovascular disease, DBP = diastolic blood pressure, eGFR = estimated glomerular filtration rate, NHANES = National Health and Nutrition Examination Survey, NPAR = neutrophil percentage-to-albumin ratio, PIR = poverty income ratio, SBP = systolic blood pressure, UACR = urinary albumin-to-creatinine ratio.

### 3.2. Kaplan–Meier curves for NPAR in predicting cardiovascular and all-cause mortality

**Figure 3.**
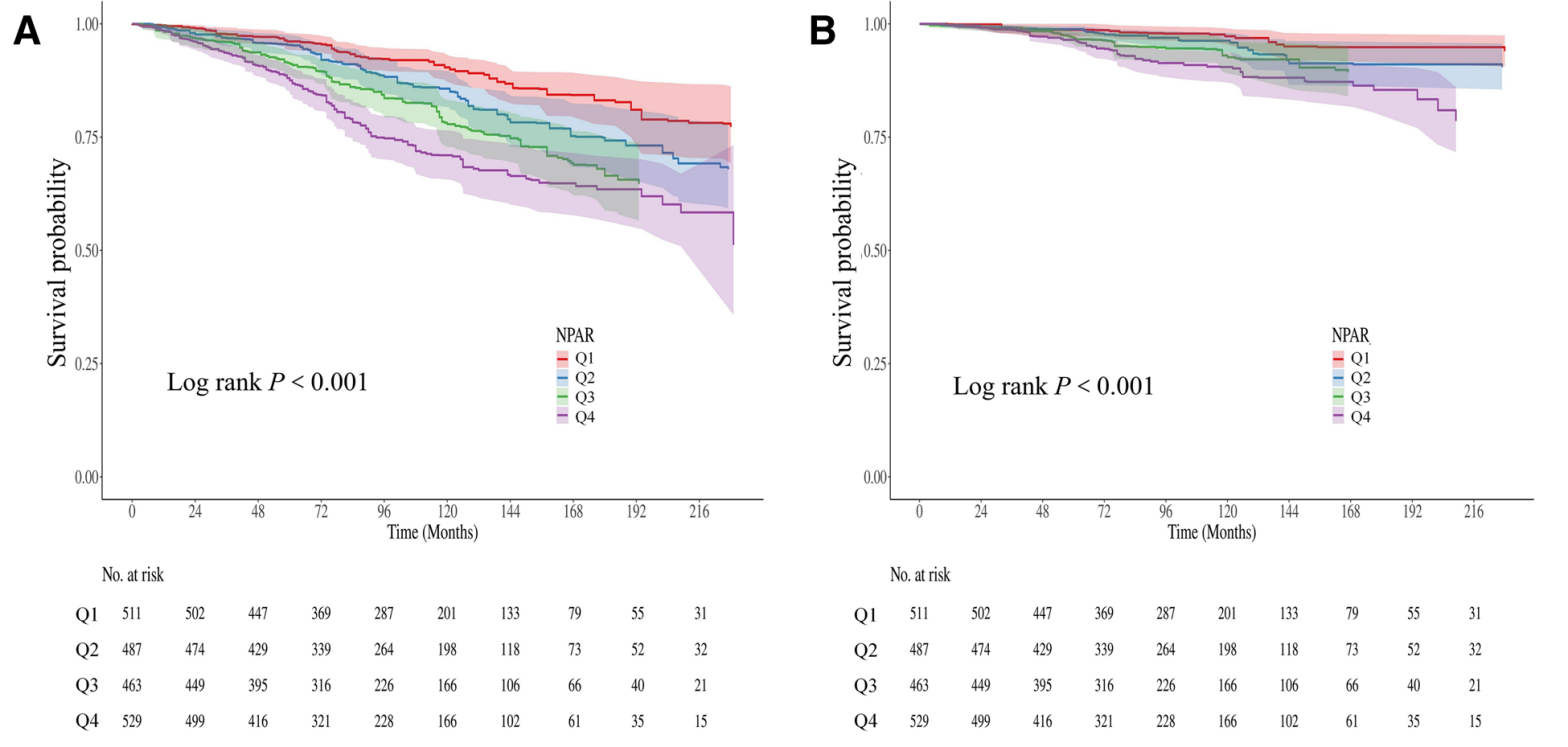
Kaplan–Meier curves for NPAR in predicting cardiovascular and all-cause mortality. (A) Kaplan–Meier curve for all-cause mortality by NPAR groups. (B) Kaplan–Meier curve for cardiovascular mortality by NPAR groups. Q1: NPAR < 12.51, Q2: 1251 ≤ NPAR < 14.27, Q3: 14.27 ≤ NPAR < 15.70, Q4: NPAR ≥ 15.70. NPAR = neutrophil percentage-to-albumin ratio.

Kaplan–Meier analysis demonstrated significant differences in survival outcomes across NPAR quartiles for both all-cause and cardiovascular mortality (log-rank test, *P* < .001 for both comparisons). Patients in the highest NPAR quartile (Q4) showed the worst survival outcomes, with only 2.8% (15/529) surviving at 216-month follow-up compared with 6.1% (31/511) in the lowest quartile (Q1; Fig. 3).

### 3.3. Predictive value of NPAR quartiles for cardiovascular and all-cause mortality risk

**Figure 4.**
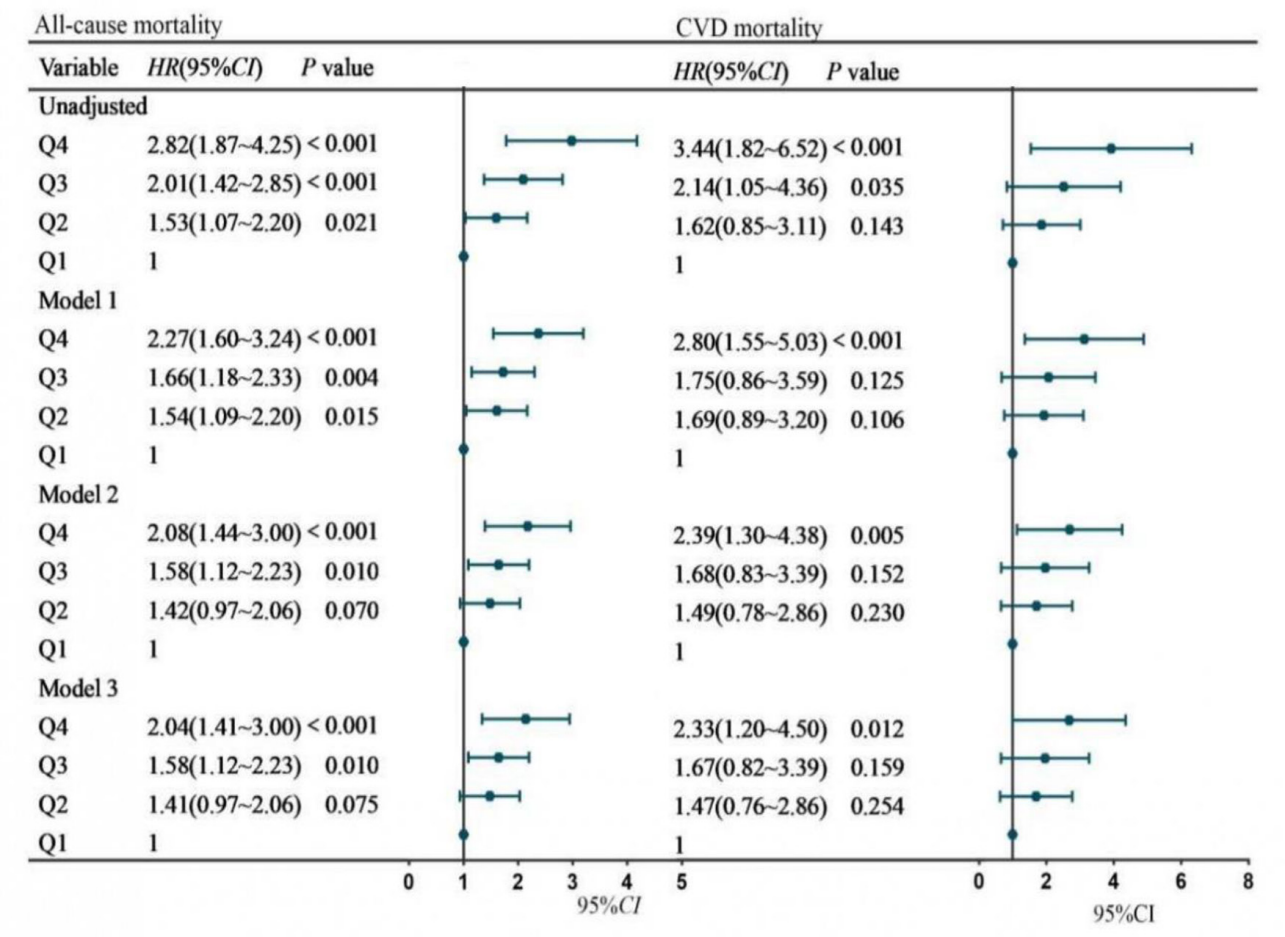
Predictive value of NPAR quartiles for cardiovascular and all-cause mortality risk. Unadjusted: unadjusted for covariates. Model 1: adjusted for confounders including age, race, smoking, and physical activity. Model 2: adjusted for confounders including age, race, smoking, physical activity, DBP, hypertension, diabetes, and CVD. Model 3: adjusted for age, race, smoking, physical activity, DBP, hypertension, diabetes, CVD, and hemoglobin. Q1: NPAR < 12.51, Q2: 12.51 ≤ NPAR < 14.27, Q3: 14.27 ≤ NPAR < 15.70, Q4: NPAR ≥ 15.70. CI = confidence interval, CVD = cardiovascular disease, DBP = diastolic blood pressure, HR = hazard ratio, NPAR = neutrophil percentage-to-albumin ratio.

Cox proportional hazards regression models were used to analyze the predictive value of quartiles, with adjustments for potential confounders, including age, race, smoking, physical activity, DBP, hypertension, diabetes, CVD, and hemoglobin. Using Q1 (lowest NPAR quartile) as the reference group, the adjusted Cox regression analysis for all-cause mortality revealed significantly higher risks in Q4 (HR = 2.04, 95% CI = 1.41–3.00, *P* < .001) and Q3 (HR = 1.58, 95% CI = 1.12–2.23, *P* = .01) compared with Q1. Similarly, in the Cox regression model for cardiovascular mortality, significantly added risks were observed in Q4 (HR = 2.33, 95% CI = 1.20–4.50, *P* = .012) compared with Q1 (Fig. 4).

### 3.4. *Dose–response relationship assessed by restricted cubic splines*

**Figure 5.**
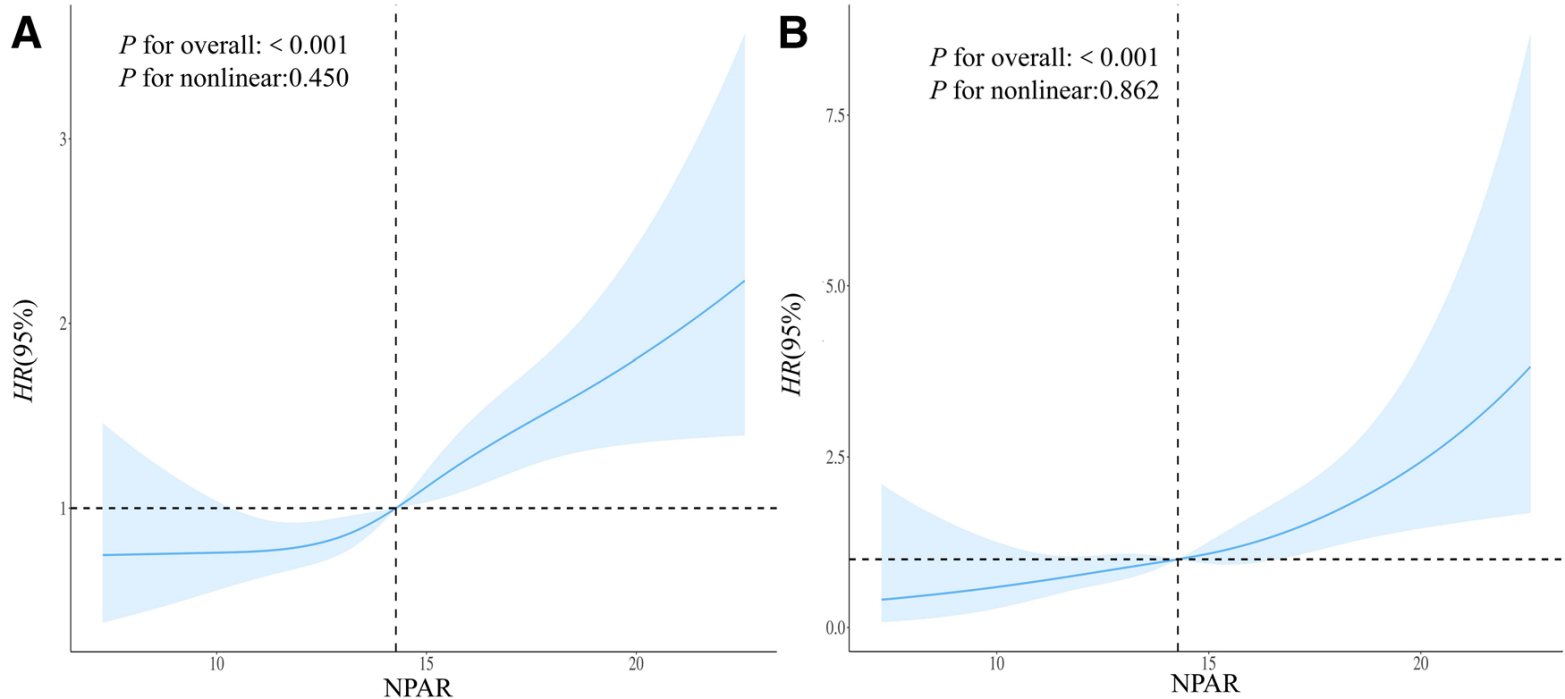
Dose–response relationship assessed by restricted cubic splines. (A) Linear relationship between NPAR and all-cause mortality. (B) Linear relationship between NPAR and all-cause mortality, adjusted for age, race, smoking, physical activity, DBP, hypertension, diabetes, CVD, and hemoglobin. CVD = cardiovascular disease, DBP = diastolic blood pressure, HR = hazard ratio, NPAR = neutrophil percentage-to-albumin ratio.

Restricted cubic spline analyses revealed significant linear associations between NPAR levels and clinical outcomes after multivariable adjustment (*P* < .001; Fig. 5).

### 3.5. Subgroup and interaction analyses of NPAR and mortality outcomes

**Figure 6.**
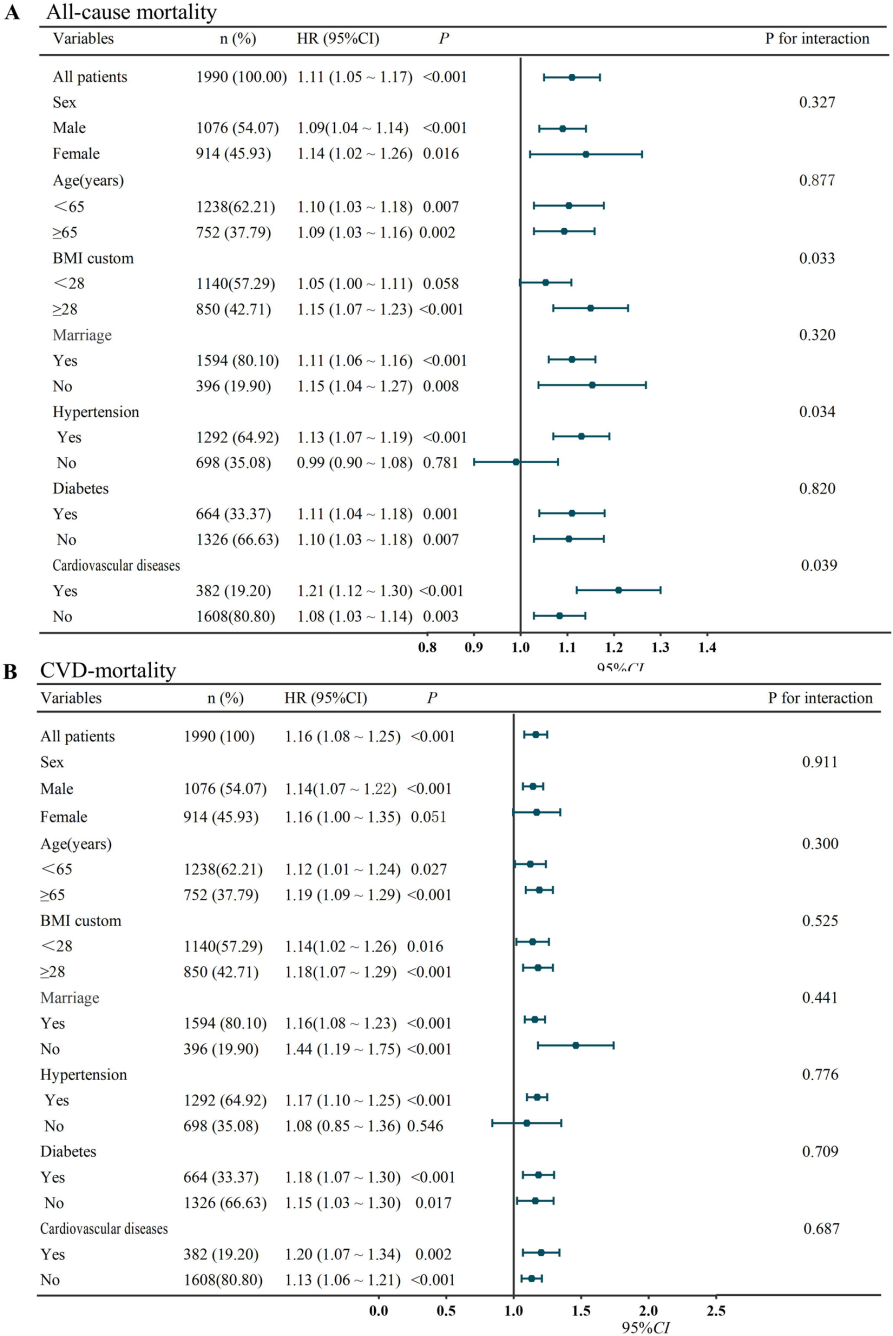
Subgroup analysis and interaction analysis. The logistic regression model was adjusted for confounders including age, race, smoking, physical activity, DBP, hypertension, diabetes, CVD, and hemoglobin. BMI = body mass index, CI = confidence interval, CVD = cardiovascular disease, DBP = diastolic blood pressure, HR = hazard ratio.

In this cohort of 1990 patients, each unit increase in NPAR level was significantly associated with increased risks of both all-cause mortality (adjusted HR = 1.11, 95% CI = 1.05–1.17, *P* < .001) and cardiovascular mortality (adjusted HR = 1.16, 95% CI = 1.08–1.25, *P* < .001). The proportional hazards assumption was validated: all-cause mortality: exploratory subgroup analyses suggested that the association between NPAR and all-cause mortality may be more pronounced in patients with obesity (BMI ≥28 vs <28 kg/m^2^: HR = 1.15 vs 1.05; *P* for interaction = .033), hypertension (HR = 1.13 vs 0.99 in nonhypertensives; *P* for interaction = .034), and CVD (HR = 1.21 vs 1.08 in non-CVD; *P* for interaction = .039). No significant interaction was observed in normal-weight patients (*P* = .058) or among nonhypertensives (*P* = .781); and cardiovascular mortality: nonsignificant associations were found in women (*P* = .051) and nonhypertensives (*P* = .546; Fig. 6).

### 3.6. Mediating effects of eGFR on the association between NPAR and mortality outcomes

**Figure 7.**
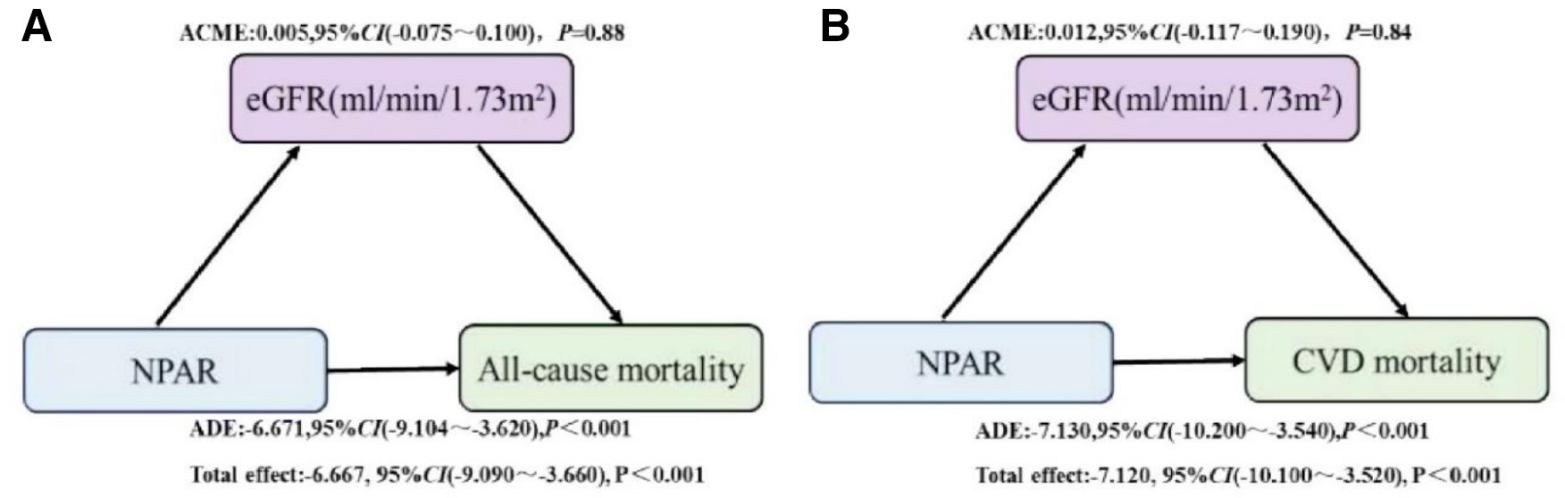
The mediating effects of eGFR on the relationship between NPAR and mortality outcomes. (A) The mediating effects of eGFR on the relationship between NPAR and all-cause mortality. (B) The mediating effects of eGFR on the relationship between NPAR and CVD mortality. (A) and (B) adjusted for confounders including age, race, smoking, physical activity, DBP, hypertension, diabetes, CVD, and hemoglobin. ACME = average causal mediation effect, ADE = average direct effect, CI = confidence interval, CVD = cardiovascular disease, DBP = diastolic blood pressure, eGFR = estimated glomerular filtration rate.

The mediation analysis revealed that in the association between NPAR and all-cause mortality, the direct effect was statistically significant (β = −6.671, 95% CI = −9.104 to −3.620, *P* < .001) with no significant mediation through eGFR (β = 0.005, 95% CI = −0.075 to –0.100, *P* = .88). Similarly, for cardiovascular mortality, NPAR exhibited a stronger direct effect (β = −7.13, 95% CI = −10.20 to −3.54, *P* < .001) with no significant mediation through eGFR (β = 0.012, 95% CI = −0.12 to –0.19, *P = *.84). All models were adjusted for age, race, smoking, physical activity, DBP, hypertension, diabetes, CVD, and hemoglobin (Fig. 7).

## 4. Discussion

The NPAR has recently emerged as a composite biomarker that integrates markers of systemic inflammation (neutrophil percentage) and nutritional status (serum albumin). Growing evidence supports its prognostic utility across various clinical conditions,^[20–22]^ but its role in CKD populations remains less well-characterized, particularly for cardiovascular mortality.^[23,24]^

Using NHANES 1999 to 2018 data, we analyzed the association between NPAR and mortality outcomes in 1990 CKD patients. The results showed that elevated NPAR levels were significantly associated with adverse outcomes in CKD patients. Patients in the highest NPAR quartile (Q4) had the lowest survival rates, with significantly higher all-cause and cardiovascular mortality than those in Q1. Multivariable Cox regression confirmed NPAR as an independent predictor of mortality, with a dose–response relationship. Exploratory subgroup analyses suggested that NPAR may have predictive value in obese, hypertensive, and CVD patients.

CKD patients commonly exhibit a low-grade chronic inflammatory state characterized by elevated pro-inflammatory cytokines (e.g., interleukin [IL]-6, tumor necrosis factor-alpha [TNF-α]) and activation of the innate immune system.^[25–28]^ Neutrophils, as key effectors of inflammation, exacerbate vascular endothelial injury and atherosclerosis through the release of reactive oxygen species, neutrophil extracellular traps (NETs), and proinflammatory mediators (e.g., IL-8, high mobility group box 1).^[29]^ Recent studies suggest that elevated neutrophil percentages in CKD patients are linked to a microinflammatory environment, potentially promoting renal fibrosis, and cardiovascular complications.^[30,31]^ The observed association between elevated NPAR and increased mortality in this study underscores the critical role of neutrophil-mediated inflammation in adverse CKD outcomes. Hypoalbuminemia, a hallmark of protein-energy wasting in CKD, arises through multiple mechanisms: inflammation suppresses hepatic albumin synthesis: proinflammatory cytokines (e.g., IL-6) downregulate hepatic albumin messenger ribonucleic acid expression^[32]^; urinary protein loss and increased catabolism: particularly in nephrotic syndrome or diabetic kidney disease, albumin is lost in urine while hypermetabolic states accelerate protein breakdown.^[33]^ Recent studies highlight that hypoalbuminemia not only reflects nutritional status but is also closely tied to oxidative stress, endothelial dysfunction, and impaired immune function.^[34,35]^ In this study, patients in Q4 had the lowest albumin levels and the poorest survival, emphasizing the nutrition-inflammation vicious cycle in CKD prognosis.

Emerging evidence continues to refine our understanding of the MIA syndrome’s pathophysiological cascade in CKD. Beyond the classical inflammation-malnutrition axis, translational research has uncovered novel mechanisms of multi-organ crosstalk (e.g., gut-kidney axis dysregulation, NETs-mediated vascular calcification).^[36–39]^ These discoveries provide potential therapeutic targets for precision intervention in MIA syndrome. Single-cell RNA sequencing analysis revealed that during the transition from acute kidney injury to CKD, Mincle^high^ neutrophils (Neu1 subset) serve as a “senescent” pro-inflammatory subset, driving the formation of a renal inflammatory microenvironment through sustained secretion of inflammatory factors such as TNF-α. Spatial transcriptomic analysis further demonstrated that the enrichment of Mincle^high^ myeloid cells in the renal outer medullary stripe region exhibited high co-localization with fibrotic areas (Acta2^+^), confirming that neutrophil-mediated inflammatory responses directly participate in the pathological progression of perivascular fibrosis.^[40]^ On the basis of current literature, the gut-kidney axis has emerged as a critical pathway in systemic inflammation. Uremia-induced gut dysbiosis leads to a significant reduction in butyrate-producing bacteria (e.g., *Faecalibacterium prausnitzii*, *Roseburia* spp.), compromising intestinal barrier integrity and increasing intestinal permeability (the “leaky gut” phenomenon). Consequently, this results in a chronic pro-inflammatory state characterized by elevated cytokines such as TNF-α and IL-6. This cascade not only exacerbates renal inflammation but also contributes to the progression of CKD and associated systemic complications.^[41]^ The vascular calcification process in MIA syndrome now appears more complex than previously recognized. Chronic inflammatory states exacerbate endothelial dysfunction through oxidative stress and pro-inflammatory cytokines (e.g., IL-6, TNF-α), thereby promoting atherosclerosis. Malnutrition reduces anti-inflammatory proteins (such as fetuin-A) and vitamin K-dependent proteins (e.g., matrix Gla protein), impairing the inhibition of vascular calcification and accelerating arterial stiffening. Hyperphosphatemia and calcium-phosphate metabolism disorders induce the osteogenic transformation of vascular smooth muscle cells, further driving vascular calcification. These factors interact synergistically, forming a vicious cycle that ultimately leads to a significant increase in cardiovascular risk.^[42,43]^ Targeted interventions addressing inflammation, nutritional imbalances, and vascular calcification may represent pivotal strategies for improving clinical outcomes.^[44,45]^ These findings mechanistically support our observation that NPAR integrates both inflammatory (NETosis) and nutritional (albumin deficiency) drivers of vascular pathology.

These results are consistent with contemporary evidence regarding the prognostic significance of inflammatory markers (e.g., C-reactive protein) and nutritional indicators (e.g., albumin) in CKD patients.^[46–49]^ However, the integrative nature of NPAR appears to offer enhanced risk stratification capacity. Notably, the observed association remains independent of eGFR levels, indicating that NPAR’s prognostic utility may extend beyond renal function parameters.

Despite these contributions, several limitations warrant consideration: the cross-sectional design of NHANES data precludes definitive causal inferences; prospective cohort studies are needed to validate NPAR’s predictive value; residual confounding: although multiple covariates were adjusted for, unmeasured confounders (e.g., medication use, dietary patterns) may persist; lack of NPAR standardization: Currently, no universal clinical cutoff exists for NPAR; this study used quartile-based classification, and future studies should establish optimal thresholds in larger populations; and external validation is lacking: These findings require replication in other populations (e.g., different ethnicities or regions). Future research directions include: conducting multicenter prospective studies to examine dynamic NPAR changes and their relationship with outcomes; integrating other inflammatory and nutritional markers (e.g., IL-6, prealbumin) to develop more comprehensive prediction models; and exploring NPAR’s potential role in guiding clinical interventions (e.g., anti-inflammatory or nutritional support therapies). In addition, the subgroup findings, particularly those with marginal *P* interaction values, should be interpreted as hypothesis-generating and require validation in independent cohorts.

## 5. Conclusion

This nationally representative cohort study demonstrates that NPAR is an independent predictor of all-cause and cardiovascular mortality in CKD patients, with particularly strong predictive value in obese, hypertensive, and CVD patients. By integrating inflammatory and nutritional status, NPAR provides a simple and reliable biomarker for risk stratification in CKD. Despite its limitations, this study lays the groundwork for future exploration of NPAR’s clinical utility and underscores the importance of comprehensive management targeting inflammation and malnutrition to improve CKD outcomes.

## Acknowledgments

We thank the National Health and Nutrition Examination Surveys for providing the data. We also acknowledge the assistance of DeepSeek in language polishing and refinement of the manuscript.

## Author contributions

**Conceptualization:** Rong Ni, Zhijian Zhang, Weiwei Li.

**Data curation:** Rong Ni, Yusheng Xu, Ci Sun.

**Formal analysis:** Rong Ni, Haixia Zhang, Linsen Jiang.

**Investigation:** Rong Ni, Yusheng Xu, Ci Sun.

**Methodology:** Rong Ni, Haixia Zhang, Linsen Jiang.

**Supervision:** Zhijian Zhang, Weiwei Li.

**Writing – original draft:** Rong Ni.

**Writing – review & editing:** Haixia Zhang, Linsen Jiang, Zhijian Zhang, Weiwei Li.

## References

[1] GBD Chronic Kidney Disease Collaboration. Global, regional, and national burden of chronic kidney disease, 1990–2017: a systematic analysis for the Global Burden of Disease Study 2017. Lancet. 2020;395:709–33. 32061315 10.1016/S0140-6736(20)30045-3 PMC7049905

[2] Matsushita K Ballew SH Wang AY Kalyesubula R Schaeffner E Agarwal R. Epidemiology and risk of cardiovascular disease in populations with chronic kidney disease. Nat Rev Nephrol. 2022;18:696–707. 36104509 10.1038/s41581-022-00616-6

[3] Thompson S James M Wiebe N. Cause of death in patients with reduced kidney function. J Am Soc Nephrol. 2015;26:2504–11. 25733525 10.1681/ASN.2014070714 PMC4587695

[4] Geng T Xu W Gao H. Relationship between control of cardiovascular risk factors and chronic kidney disease progression, cardiovascular disease events, and mortality in Chinese adults. J Am Coll Cardiol. 2024;84:1313–24. 39322325 10.1016/j.jacc.2024.06.041

[5] Kalantar-Zadeh K Stenvinkel P Pillon L Kopple JD. Inflammation and nutrition in renal insufficiency. Adv Ren Replace Ther. 2003;10:155–69. 14708070 10.1053/j.arrt.2003.08.008

[6] Graterol Torres F Molina M Soler-Majoral J. Evolving concepts on inflammatory biomarkers and malnutrition in chronic kidney disease. Nutrients. 2022;14:4297. 36296981 10.3390/nu14204297 PMC9611115

[7] Bonanni A Mannucci I Verzola D. Protein-energy wasting and mortality in chronic kidney disease. Int J Environ Res Public Health. 2011;8:1631–54. 21655142 10.3390/ijerph8051631 PMC3108132

[8] Nowak KL Chonchol M. Does inflammation affect outcomes in dialysis patients? Semin Dial. 2018;31:388–97. 29513906 10.1111/sdi.12686 PMC6035073

[9] Rambod M Kovesdy CP Kalantar-Zadeh K. Malnutrition-inflammation score for risk stratification of patients with CKD: is it the promised gold standard? Nat Clin Pract Nephrol. 2008;4:354–5. 18523431 10.1038/ncpneph0834

[10] Hu C He Y Li J. Association between neutrophil percentage-to-albumin ratio and 28-day mortality in Chinese patients with sepsis. J Int Med Res. 2023;51:3000605231178512. 37314249 10.1177/03000605231178512 PMC10291015

[11] Gong Y Li D Cheng B Ying B Wang B. Increased neutrophil percentage-to-albumin ratio is associated with all-cause mortality in patients with severe sepsis or septic shock. Epidemiol Infect. 2020;148:e87. 32238212 10.1017/S0950268820000771 PMC7189348

[12] Seydel GS Gunturk I Akkaya H Gunturk EE. The relationship between the new inflammatory markers and disease severity in patients with acute coronary syndrome. Acta Cardiol. 2024;79:778–86. 39287020 10.1080/00015385.2024.2403933

[13] Lin Y Lin Y Yue J Zou Q. The neutrophil percentage-to-albumin ratio is associated with all-cause mortality in critically ill patients with acute myocardial infarction. BMC Cardiovasc Disord. 2022;22:115. 35300600 10.1186/s12872-022-02559-z PMC8932161

[14] Ko CA Fang KH Tsai MS. Prognostic value of neutrophil percentage-to-albumin ratio in patients with oral cavity cancer. Cancers. 2022;14:4892. 36230814 10.3390/cancers14194892 PMC9564168

[15] Liang H Pan K Wang J Lin J. Association between neutrophil percentage-to-albumin ratio and breast cancer in adult women in the US: findings from the NHANES. Front Nutr. 2025;12:1533636. 40357031 10.3389/fnut.2025.1533636 PMC12066505

[16] Ferro M Babă DF de Cobelli O. Neutrophil percentage-to-albumin ratio predicts mortality in bladder cancer patients treated with neoadjuvant chemotherapy followed by radical cystectomy. Future Sci OA. 2021;7:FSO709. 34258022 10.2144/fsoa-2021-0008 PMC8256323

[17] Wang X Zhang Y Wang Y. The neutrophil percentage-to-albumin ratio is associated with all-cause mortality in patients with chronic heart failure. BMC Cardiovasc Disord. 2023;23:568. 37980510 10.1186/s12872-023-03472-9 PMC10657562

[18] Levey AS Stevens LA Schmid CH. A new equation to estimate glomerular filtration rate. Ann Intern Med. 2009;150:604–12. 19414839 10.7326/0003-4819-150-9-200905050-00006 PMC2763564

[19] Andrassy KM. Comments on “KDIGO 2012 clinical practice guideline for the evaluation and management of chronic kidney disease”. Kidney Int. 2013;84:622–3. 10.1038/ki.2013.243 23989362

[20] Liu CF Chien LW. Predictive role of neutrophil-percentage-to-albumin ratio (NPAR) in nonalcoholic fatty liver disease and advanced liver fibrosis in nondiabetic US adults: evidence from NHANES 2017–2018. Nutrients. 2023;15:1892. 37111111 10.3390/nu15081892 PMC10141547

[21] He X Dai F Zhang X Pan J. The neutrophil percentage-to-albumin ratio is related to the occurrence of diabetic retinopathy. J Clin Lab Anal. 2022;36:e24334. 35285099 10.1002/jcla.24334 PMC8993596

[22] Lan CC Su WL Yang MC Chen SY Wu YK. Predictive role of neutrophil-percentage-to-albumin, neutrophil-to-lymphocyte and eosinophil-to-lymphocyte ratios for mortality in patients with COPD: evidence from NHANES 2011–2018. Respirology. 2023;28:1136–46. 37655985 10.1111/resp.14589

[23] Li J Xiang T Chen X Fu P. Neutrophil-percentage-to-albumin ratio is associated with chronic kidney disease: evidence from NHANES 2009–2018. PLoS One. 2024;19:e0307466. 39102412 10.1371/journal.pone.0307466 PMC11299806

[24] Zhao M Huang X Zhang Y Wang Z Zhang S Peng J. Predictive value of the neutrophil percentage-to-albumin ratio for coronary atherosclerosis severity in patients with CKD. BMC Cardiovasc Disord. 2024;24:277. 38807036 10.1186/s12872-024-03896-x PMC11134736

[25] Kadatane SP Satariano M Massey M Mongan K Raina R. The role of inflammation in CKD. Cells. 2023;12:1581. 37371050 10.3390/cells12121581 PMC10296717

[26] Hu Y Hao F An Q Jiang W. Immune cell signatures and inflammatory mediators: unraveling their genetic impact on chronic kidney disease through Mendelian randomization. Clin Exp Med. 2024;24:94. 38703294 10.1007/s10238-024-01341-z PMC11069478

[27] Tinti F Lai S Noce A. Chronic kidney disease as a systemic inflammatory syndrome: update on mechanisms involved and potential treatment. Life (Basel). 2021;11:419. 34063052 10.3390/life11050419 PMC8147921

[28] Wu J Guo N Chen X Xing C. Coexistence of micro-inflammatory and macrophage phenotype abnormalities in chronic kidney disease. Int J Clin Exp Pathol. 2020;13:317–23. 32211115 PMC7061787

[29] Wang H Kim SJ Lei Y. Neutrophil extracellular traps in homeostasis and disease. Signal Transduct Target Ther. 2024;9:235. 39300084 10.1038/s41392-024-01933-x PMC11415080

[30] Humphreys BD. Mechanisms of renal fibrosis. Annu Rev Physiol. 2018;80:309–26. 29068765 10.1146/annurev-physiol-022516-034227

[31] Jankowski J Floege J Fliser D Böhm M Marx N. Cardiovascular disease in chronic kidney disease: pathophysiological insights and therapeutic options. Circulation. 2021;143:1157–72. 33720773 10.1161/CIRCULATIONAHA.120.050686 PMC7969169

[32] Kaysen GA Dubin JA Müller HG Rosales L Levin NW Mitch WE. Inflammation and reduced albumin synthesis associated with stable decline in serum albumin in hemodialysis patients. Kidney Int. 2004;65:1408–15. 15086482 10.1111/j.1523-1755.2004.00520.x

[33] Matyjek A Literacki S Niemczyk S Rymarz A. Protein energy-wasting associated with nephrotic syndrome – the comparison of metabolic pattern in severe nephrosis to different stages of chronic kidney disease. BMC Nephrol. 2020;21:346. 32795277 10.1186/s12882-020-02003-4 PMC7427894

[34] de Castro LL de Carvalho e Martins MC Garcez AM. Hypoalbuminemia and oxidative stress in patients on renal hemodialysis program. Nutr Hosp. 2014;30:952–9. 25335687 10.3305/nh.2014.30.4.7667

[35] Arques S. Human serum albumin in cardiovascular diseases. Eur J Intern Med. 2018;52:8–12. 29680174 10.1016/j.ejim.2018.04.014

[36] Douvris A Viñas JL Gutsol A Zimpelmann J Burger D Burns KD. miR-486-5p protects against rat ischemic kidney injury and prevents the transition to chronic kidney disease and vascular dysfunction. Clin Sci (Lond). 2024;138:599–614. 38739452 10.1042/CS20231752 PMC11130553

[37] Kim JK Park MJ Lee HW. The relationship between autophagy, increased neutrophil extracellular traps formation and endothelial dysfunction in chronic kidney disease. Clin Immunol. 2018;197:189–97. 30296592 10.1016/j.clim.2018.10.003

[38] Amini Khiabani S Asgharzadeh M Samadi Kafil H. Chronic kidney disease and gut microbiota. Heliyon. 2023;9:e18991. 37609403 10.1016/j.heliyon.2023.e18991 PMC10440536

[39] Hu K Zhong L Lin W. Pathogenesis-guided rational engineering of nanotherapies for the targeted treatment of abdominal aortic aneurysm by inhibiting neutrophilic inflammation. ACS Nano. 2024;18:6650–72. 38369729 10.1021/acsnano.4c00120

[40] Wang C Zhang Y Shen A. Mincle receptor in macrophage and neutrophil contributes to the unresolved inflammation during the transition from acute kidney injury to chronic kidney disease. Front Immunol. 2024;15:1385696. 38770013 10.3389/fimmu.2024.1385696 PMC11103384

[41] Di Vincenzo F Del Gaudio A Petito V Lopetuso LR Scaldaferri F. Gut microbiota, intestinal permeability, and systemic inflammation: a narrative review. Intern Emerg Med. 2024;19:275–93. 37505311 10.1007/s11739-023-03374-w PMC10954893

[42] Düsing P Zietzer A Goody PR. Vascular pathologies in chronic kidney disease: pathophysiological mechanisms and novel therapeutic approaches. J Mol Med (Berl). 2021;99:335–48. 33481059 10.1007/s00109-021-02037-7 PMC7900031

[43] Gungor O Kocyigit I Yilmaz MI Sezer S. Role of vascular calcification inhibitors in preventing vascular dysfunction and mortality in hemodialysis patients. Semin Dial. 2018;31:72–81. 28608927 10.1111/sdi.12616

[44] Raggi P Bellasi A Bushinsky D. Slowing progression of cardiovascular calcification with SNF472 in patients on hemodialysis: results of a randomized Phase 2b study. Circulation. 2020;141:728–39. 31707860 10.1161/CIRCULATIONAHA.119.044195

[45] Lee SM An WS. Supplementary nutrients for prevention of vascular calcification in patients with chronic kidney disease. Korean J Intern Med. 2019;34:459–69. 31048656 10.3904/kjim.2019.125 PMC6506750

[46] Lee C Park KH Joo YS. Low high-sensitivity C-reactive protein level in Korean patients with chronic kidney disease and its predictive significance for cardiovascular events, mortality, and adverse kidney outcomes: results from KNOW-CKD. J Am Heart Assoc. 2020;9:e017980. 33092438 10.1161/JAHA.120.017980 PMC7763415

[47] Tokuda K Tanaka A Tobe A. Impact of C-reactive protein on long-term cardiac events in stable coronary artery disease patients with chronic kidney disease. J Atheroscler Thromb. 2023;30:1635–43. 36908149 10.5551/jat.64047 PMC10627763

[48] Huang F Fan J Wan X. The association between blood albumin level and cardiovascular complications and mortality risk in ICU patients with CKD. BMC Cardiovasc Disord. 2022;22:322. 35850629 10.1186/s12872-022-02763-x PMC9295487

[49] Kikuchi H Kanda E Mandai S. Combination of low body mass index and serum albumin level is associated with chronic kidney disease progression: the chronic kidney disease-research of outcomes in treatment and epidemiology (CKD-ROUTE) study. Clin Exp Nephrol. 2017;21:55–62. 26920126 10.1007/s10157-016-1251-2

